# Impact of COVID-19 on urgent care diagnoses and the new AXR metric

**DOI:** 10.1017/ash.2024.62

**Published:** 2024-04-18

**Authors:** Adam L. Hersh, Edward A. Stenehjem, Nora Fino, Emily S. Spivak

**Affiliations:** 1 Department of Pediatrics, Division of Infectious Diseases, University of Utah, Salt Lake City, UT, USA; 2 Department of Medicine, Division of Infectious Diseases, University of Colorado School of Medicine, Aurora, CO, USA; 3 Urgent Care, University of Utah, Salt Lake City, UT, USA; 4 Department of Internal Medicine, Division of Epidemiology, University of Utah, Salt Lake City, UT, USA; 5 Department of Internal Medicine, Division of Infectious Diseases, University of Utah, Salt Lake City, UT, USA

## Abstract

We examined the antibiotic prescribing rate for respiratory diagnoses (AXR) before and after onset of the COVID-19 pandemic in urgent care clinics. At the onset, AXR declined substantially due to changes in case mix. Using AXR as a stewardship metric requires monitoring of changes in case mix.

## Introduction

The antibiotic utilization for respiratory conditions (AXR), defined as the percentage of respiratory encounters prescribed an antibiotic, is an antibiotic stewardship metric and was introduced as a Healthcare Effectiveness Data and Information Set (HEDIS) measure in 2022.^1^ This metric and related metrics have been featured in studies of antibiotic stewardship interventions with reductions corresponding to successful implementation.^2–4^ Advantages of this metric include that it encompasses a broad range of common clinical encounters seen in outpatient care and is less vulnerable to differences in coding practices or code shifting since all respiratory diagnoses are considered together. However, case-mix differences in the types of respiratory conditions cared for between settings (eg, urgent care versus emergency department) or that may emerge over time within the same setting have the potential to directly impact this metric in ways that could complicate interpretation of temporal changes.

The onset of the coronavirus disease 2019 (COVID-19) pandemic in March 2020 initially reduced healthcare utilization but ultimately introduced a high number of viral illnesses for which antibiotics are not recommended.^5,6^ The impact of this shift in case-mix on respiratory diagnoses and the AXR has not been explored.

## Methods

This was a retrospective cohort of encounters for respiratory diagnoses in the University of Utah Urgent Care clinic system from March 2019 to March 2022. This included nine clinics staffed predominantly by advanced practice clinicians. Most encounters are in-person but telemedicine visits were included as well and staffed by the same clinicians. All clinics had molecular testing for COVID-19. The study was approved by the University of Utah Institutional Review Board.

We examined antibiotic prescribing rates for respiratory diagnoses in these clinics during two periods: pre-pandemic (March 2019–February 2020) and pandemic (March 2020–March 2022). We also divided the pandemic period further to reflect the first and second waves (March–December 2020, Jan 2021–March 2022). Respiratory diagnoses were identified using International Classification of Diseases, Tenth Revision, Clinical Modification (ICD10) codes and further stratified into 3 Tiers (Tier 1: antibiotics indicated; Tier 2: antibiotics sometimes indicated; Tier 3: antibiotics not indicated) and further into individual diagnosis categories within tiers based on a previously published approach.^3^ Tier 1 categories included: pneumonia, streptococcal pharyngitis, and mastoiditis; Tier 2 categories included: acute otitis media, pharyngitis and tonsilitis, and sinusitis; Tier 3 categories included: asthma, bronchitis, COVID-19, cough, influenza, laryngitis and tonsilitis, rhinitis, upper respiratory tract infection and other respiratory conditions. We examined trends in antibiotic prescribing across these periods including the percentage of respiratory visits prescribed antibiotics overall to approximate the AXR and by Tier and the distribution of diagnoses within Tiers. No formalized stewardship interventions were introduced during these periods.

## Results

**Figure 1.**
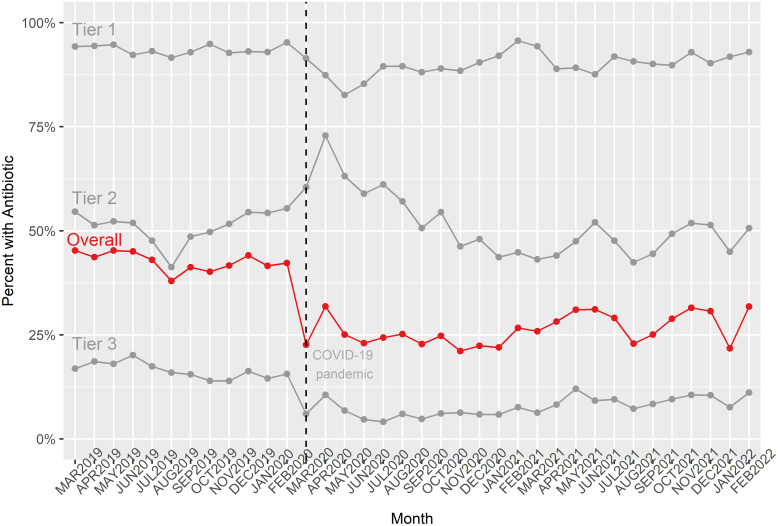
Overall monthly time trends in respiratory prescribing rate for encounters across the study period overall and by Tier. Tier 1: antibiotics indicated; Tier 2: antibiotics sometimes indicated; Tier 3: antibiotics not indicated.

There were 132,730 UC visits for respiratory diagnoses during the study. The respiratory visits included 44,807 during the pre-pandemic and 87,923 during the pandemic periods. In aggregate, the AXR declined from 42.8% (19,156/44,807) during the pre-pandemic period to 26.3% (23,111/87,923) during the pandemic period (Table 1). When examined by waves, the largest reduction occurred during the first wave (23.7%) and increased somewhat during the second wave (27.5%) (Figure 1).

**Table 1.** Summary of all respiratory encounters in the study period. Pre-pandemic was March 2019–February 2020 and pandemic was March 2020–March 2022

	Pre-pandemic		Pandemic	
	Overall N (% of all encounters)	N with Abx (% of all encounters with Abx)	N (% of all encounters)	N with Abx (% of all encounters with Abx)
Overall	44,807	19,156 (42.8%)	87,923	23,111 (26.3%)
By Tier:				
1	5,869 (13.1%)	5,496 (93.6%)	3,844 (4.4%)	3,480 (90.5%)
2	20,536 (45.8%)	10,693 (52.1%)	30,945 (35.2%)	15,441 (49.9%)
3	18,402 (41.1%)	2,967 (16.1%)	53,134 (60.4%)	4,190 (7.9%)
By diagnosis				
Asthma	823 (1.8%)	60 (7.3%)	1,584 (1.8%)	76 (4.8%)
Bronchitis	1,307 (2.9%)	520 (39.8%)	964 (1.1%)	397 (41.2%)
COVID-19	0 (0.0%)	0 (0.0%)	12,169 (13.8%)	232 (1.9%)
Cough	3,277 (7.3%)	860 (26.2%)	9,632 (11.0%)	1,120 (11.6%)
Epiglottitis	1 (0.0%)	1 (100.0%)	1 (0.0%)	0 (0%)
Influenza	344 (0.8%)	16 (4.7%)	120 (0.1%)	3 (2.5%)
Laryngitis/Tracheitis	739 (1.6%)	19 (2.6%)	873 (1.0%)	15 (1.7%)
Mastoiditis	1 (0.0%)	0 (0.0%)	4 (0.0%)	1 (25.0%)
Otitis Media	4,172 (9.3%)	4,044 (96.9%)	5,102 (5.8%)	4,929 (96.6%)
Pharyngitis and Tonsillitis	11,170 (24.9%)	1,989 (17.8%)	17,996 (20.5%)	3,555 (19.8%)
Pneumonia	1,157 (2.6%)	1,047 (90.5%)	1,044 (1.2%)	876 (83.9%)
Respiratory Other	3,973 (8.9%)	692 (17.4%)	10,225 (11.6%)	1,152 (11.3%)
Rhinitis	245 (0.5%)	3 (1.2%)	553 (0.6%)	13 (2.4%)
Serous Otitis Media	764 (1.7%)	522 (68.3%)	1,023 (1.2%)	634 (62.0%)
Sinusitis	4,919 (11.0%)	4,455 (90.6%)	7,488 (8.5%)	6,758 (90.3%)
Streptococcal Pharyngitis	4,646 (10.4%)	4,415 (95.0%)	2,697 (3.1%)	2,554 (94.7%)
URI NOS	7,269 (16.2%)	513 (7.1%)	16,448 (18.7%)	796 (4.8%)

The distribution of respiratory diagnoses (by Tiers and specific diagnoses) and antibiotic prescribing is shown in Table 1. The percentage of Tier 1 diagnoses decreased from 13.1% to 4.4%, the percentage of Tier 2 diagnoses decreased from 45.8% to 35.2% and the percentage of Tier 3 diagnoses increased from 41.1% to 60.4% (Table 1). Antibiotic prescribing within Tier changed minimally for Tier 1 and Tier 2 (and more substantially for Tier 3 diagnoses (16.1% to 7.9%) (Table 1, Figure 1).

When examining specific diagnoses, the most notable changes occurred within Tier 3 diagnoses. The percentage of all respiratory diagnoses for COVID-19 increased from 0% to 13.8% (12,169 total) of all encounters (Table 1). Only 1.9% of these encounters were prescribed an antibiotic (Table 1).

## Discussion

We found that the AXR metric declined substantially at the onset of the COVID-19 pandemic and was sustained at a lower level for a prolonged period. The magnitude of this change resembled the reductions previously reported following stewardship interventions in outpatient settings.^3^ However, other than general education that COVID-19 does not generally require antibiotic therapy, no explicit stewardship interventions were implemented during this period in these urgent care clinics. Our analysis revealed that the major cause of the reduction in the AXR metric was due to an increase in prevalence of Tier 3 conditions which were prescribed antibiotics at relatively low rates, primarily COVID-19. These findings demonstrate the importance of careful monitoring of diagnostic distributions when tracking AXR as a metric.

Prior studies have used AXR as a primary outcome to study the impact of stewardship interventions in outpatient settings.^3^ One study in urgent care demonstrated the utility of this metric by detecting a sudden increase in the AXR which enabled identifying the cause as an increased prescribing rate for pharyngitis due to the temporary elimination of rapid testing.^7^ However, other than this temporary increase in prescribing, the onset of the COVID-19 pandemic did not lead to an abrupt decline in AXR in this health system likely due to a preceding stewardship intervention that had already reduced prescribing, especially for Tier 3 conditions, to a low level.^3^ This may have been enhanced in part due to availability of point-of-care testing and education about not prescribing antibiotics for COVID-19. Other studies including in an urgent care system have shown similar trends in antibiotic prescribing rates for respiratory conditions, although not explicitly with AXR as a pre-specified metric.^2,4^

Although the most notable changes contributing to the decline in AXR were the increase in Tier 3 diagnoses (primarily COVID-19) and reduction in the percentage that received antibiotics, small reductions in prescribing for Tier 1 and 2 diagnoses also occurred. These favorable changes suggest that this metric may have value as a proxy for rates of unnecessary prescribing.

This study was focused on a network of urgent care clinics at a single institution. Although not generalizable, a valuable application of the AXR metric locally will be inter-clinician comparisons and benchmarking. Future applications of the AXR metric will likely have the purpose of comparing prescribing between clinics or systems across disparate geographic regions and clinic types. Recognizing the importance of case-mix differences that exist between clinical settings will be crucial to ensure that benchmarking on a larger scale is appropriate.

Our study has limitations. We used ICD10 codes which can lead to misclassification and may differ from the exact codes used by HEDIS which are not publicly available. Although no active stewardship interventions occurred, some of the AXR decline could have been due to successful education that antibiotics are not required for COVID-19 which caused many illnesses during this period.

In conclusion, although the AXR metric is valuable for tracking purposes in outpatient stewardship, we found a significant change in this metric at the onset of the COVID-19 pandemic due primarily to changes in case-mix with the introduction of a larger percentage of viral infections diagnosed in urgent care. Outpatient stewardship programs or other organizations tracking AXR for trends over time or for comparisons between settings should take care to understand the origin of changes or differences because these can be due not only to the introduction of stewardship interventions but also changes in case-mix.
